# 3D distortion‐free, reduced FOV diffusion‐prepared gradient echo at 3 T

**DOI:** 10.1002/mrm.30357

**Published:** 2024-10-27

**Authors:** Sarah McElroy, Raphael Tomi‐Tricot, Jon Cleary, Hsern Ern Ivan Tan, Shawna Kinsella, Sami Jeljeli, Vicky Goh, Radhouene Neji

**Affiliations:** ^1^ School of Biomedical Engineering and Imaging Sciences King's College London London UK; ^2^ MR Research Collaborations Siemens Healthcare Limited Camberley UK; ^3^ Siemens Healthcare Courbevoie France; ^4^ Guy's and St Thomas' NHS Foundation Trust London UK

**Keywords:** diffusion imaging, diffusion‐prepared imaging, distortion‐free diffusion, reduced FOV diffusion, spinal cord DWI

## Abstract

**Purpose:**

To develop a 3D distortion‐free reduced‐FOV diffusion‐prepared gradient‐echo sequence and demonstrate its application in vivo for diffusion imaging of the spinal cord in healthy volunteers.

**Methods:**

A 3D multi‐shot reduced‐FOV diffusion‐prepared gradient‐echo acquisition is achieved using a slice‐selective tip‐down pulse in the phase‐encoding direction in the diffusion preparation, combined with magnitude stabilizers, centric k‐space encoding, and 2D phase navigators to correct for intershot phase errors. The accuracy of the ADC values obtained using the proposed approach was evaluated in a diffusion phantom and compared to the tabulated reference ADC values and to the ADC values obtained using a standard spin echo diffusion‐weighted single‐shot EPI sequence (DW‐SS‐EPI). Five healthy volunteers were scanned at 3 T using the proposed sequence, DW‐SS‐EPI, and a clinical diffusion‐weighted multi‐shot readout‐segmented EPI sequence (RESOLVE) for cervical spinal cord imaging. Image quality, perceived SNR, and image distortion were assessed by two expert radiologists. ADC maps were calculated, and ADC values obtained with the proposed sequence were compared to those obtained using DW‐SS‐EPI and RESOLVE.

**Results:**

Consistent ADC estimates were measured in the diffusion phantom with the proposed sequence and the conventional DW‐SS‐EPI sequence, and the ADC values were in close agreement with the reference values provided by the manufacturer of the phantom. In vivo, the proposed sequence demonstrated improved image quality, improved perceived SNR, and reduced perceived distortion compared to DW‐SS‐EPI, whereas all measures were comparable against RESOLVE. There were no significant differences in ADC values estimated in vivo for each of the sequences.

**Conclusion:**

3D distortion‐free diffusion‐prepared imaging can be achieved using the proposed sequence.

## 1 INTRODUCTION

DWI is a powerful tool for probing the microstructure to assess a range of pathologies, including stroke, tumors, and inflammatory conditions.^1^, ^2^

Conventional DWI employs a Stejskal–Tanner pulsed gradient spin echo^3^ in combination with a single‐shot EPI (SS‐EPI) readout, which is efficient and insensitive to motion.^4^ However, SS‐EPI is prone to geometrical distortion, signal loss, and blurring, which are exacerbated at higher field strengths due to increased off‐resonance and shorter $T_{2}^{*}$ relaxation times. Signal loss and image blurring are also increased with the longer readout durations required for higher spatial resolution, which is a key benefit of imaging at higher field strengths.

Scan acceleration using parallel imaging^5^, ^6^ to reduce the readout duration and the effective echo spacing can help to reduce^7^, ^8^, ^9^ but cannot fully eliminate these effects, especially for imaging regions with high susceptibility gradients^10^ such as the frontal lobes, skull base, cervical spine, and near metal implants where shimming of the static magnetic field can be very challenging.

A further reduction in the readout duration and effective echo spacing can be achieved using multi‐shot EPI, including interleaved EPI^11^, ^12^ or readout‐segmented EPI.^13^, ^14^ The main challenge introduced with these multi‐shot approaches is the intershot nonlinear phase errors due to physiological motion in the presence of strong diffusion gradients. It is therefore necessary to correct for these phase offsets before combining the acquired shots. Solutions for phase correction include phase navigators, with initial methods proposing a 1D linear phase correction using 1D navigator echoes^15^, ^16^ and the use of 2D navigators for correcting linear and constant phase offsets in k‐space.^12^ 2D nonlinear phase correction was proposed in DW‐PROPELLER^17^ and was later formulated in a general refocusing reconstruction framework.^18^ Other approaches that do not involve an explicit acquisition of a phase navigator have also been proposed. These include self‐navigated techniques that derive shot‐dependent phase maps from the acquired data,^19^, ^20^, ^21^, ^22^, ^23^, ^24^, ^25^, ^26^ navigator‐free reconstruction techniques that recover uncorrupted data via a regularization prior such as a low rank constraint^27^, ^28^, ^29^, ^30^, ^31^, ^32^, ^33^ or a deep learning–based prior,^34^, ^35^, ^36^ as well as a combination of these approaches.^37^

Another approach to reduce distortions in diffusion imaging is to use reduced FOV (RFOV) EPI techniques^38^, ^39^, ^40^ by restricting the FOV in the phase‐encoding direction with, for example, outer volume suppression or 2D RF excitation pulses. RFOV approaches offer some advantages over parallel imaging, including a simpler implementation of the phase correction (no parallel imaging reconstruction is required for the navigator shots, which would also be undersampled to obtain a phase map with sufficient resolution), no g‐factor SNR penalty, and avoiding artifacts and unwanted signals from short T_1_, high‐signal producing regions (e.g., chest and/or back fat for spinal cord imaging) outside of the anatomical region of interest (ROI).

However, distortion may still be present in areas of high susceptibility gradients. Other EPI‐based approaches^41^, ^42^, ^43^ achieve distortion‐free diffusion imaging using techniques such as point‐spread‐function EPI encoding,^44^, ^45^ which combines EPI with additional phase encoding steps; or echo planar time‐resolved imaging,^46^ which enables high acceleration of a ky‐t acquisition to reconstruct distortion‐free images at each time point. However, these methods are based on additional encoding, therefore prolonging scan times.

Other techniques have proposed using an alternative imaging readout to EPI. For example, diffusion‐preparation (DP) modules have been proposed, which can be combined with any imaging readout to completely avoid distortion even in regions of high‐susceptibility gradients.^47^ The DP module typically consists of a nonselective tip‐down pulse, followed by diffusion‐sensitizing gradients, refocusing pulses, and a nonselective tip‐up pulse, which stores the diffusion‐prepared magnetization in the longitudinal direction prior to readout. One of the main challenges with diffusion‐prepared imaging is that phase errors due to motion and eddy currents will result in spatially variable magnitude errors.^48^ Several solutions have been proposed, including preparatory gradients,^49^ motion‐compensated diffusion gradients,^50^ phase cycling of the tip‐up pulse,^48^, ^51^ and magnitude stabilizers.^52^, ^53^ Recent work suggests that magnitude stabilizers are essential for correction of motion‐related phase errors.^54^ The use of magnitude stabilizers in the DP combined with rewinder gradients in the imaging module leads to the generation of stimulated echoes, and is therefore closely related to the diffusion‐weighted STEAM (DW STEAM)^55^ and particularly to the DW turbo‐STEAM sequence,^56^, ^57^, ^58^, ^59^ which extends DW STEAM using a DP with slice‐selective 90° and 180° pulses.

DP has been combined with turbo spin echo (TSE),^60^, ^61^, ^62^, ^63^ balanced steady‐state free precession,^50^, ^64^, ^65^ and gradient‐echo (GRE) readouts.^48^, ^51^, ^53^ Each of these readouts have advantages and limitations, which need to be considered for a particular application and field strength. Whereas TSE and balanced steady‐state free precession offer higher relative SNR, GRE uses RF pulses with lower flip angles and therefore has a lower specific absorption rate (SAR) burden,^66^ which is advantageous at higher field strengths. One potential disadvantage of the GRE readout for diffusion‐prepared imaging is the introduction of T_1_ weighting, which can be minimized by employing a centric encoding.^51^ T_1_ effects can also impact on accuracy of calculated ADC values. These biases can be avoided by employing magnitude stabilizers^67^ or by employing phase cycling and ensuring the delay time before each DP is slightly greater than the T_1_, which is also the condition for optimal SNR efficiency.^48^

In this work, we present a novel technique for distortion‐free diffusion‐prepared imaging, which extends the DW turbo‐STEAM and the diffusion‐prepared stimulated echo turboFLASH^53^ sequences to a 3D multi‐shot, reduced FOV acquisition. The proposed sequence uses a multi‐shot 3D GRE readout, combined with centric encoding, magnitude stabilizers, and reduced FOV. The technique is demonstrated in a diffusion phantom and for 3 T sagittal imaging of the cervical spinal cord, which is particularly prone to distortion using a conventional SS‐EPI acquisition. Part of this work has been presented at the International Society for Magnetic Resonance in Medicine 2024 annual meeting,^68^ where a parallel and independent work pursued a similar idea.^69^

## 2 METHODS

### 2.1 Sequence design and image reconstruction

**FIGURE 1:**
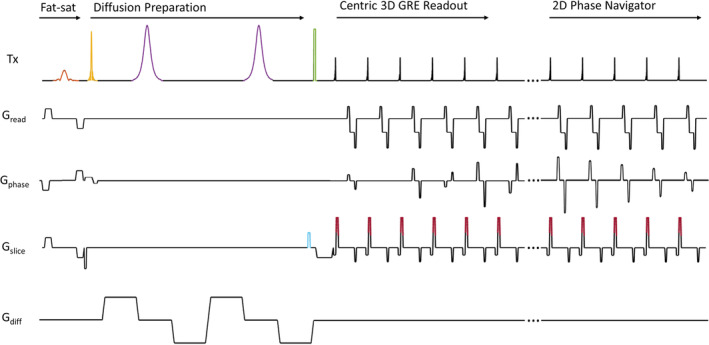
Pulse sequence diagram for 3D diffusion‐prepared gradient echo acquisition. A chemical shift‐selective fat saturation pulse (orange) precedes the tip‐down pulse (yellow), which is applied in combination with a slab‐selective gradient in the phase‐encoding direction. This is followed by a pair of bipolar diffusion gradients, each surrounding a 180° adiabatic hyperbolic secant refocusing pulse (purple). A magnitude stabilizer dephasing gradient (blue) is applied before the nonselective tip‐up pulse (green). The magnitude stabilizer re‐phasing gradient (red) is incorporated in the slice rewinder gradient as an additional moment after each readout excitation pulse.

**FIGURE 2:**
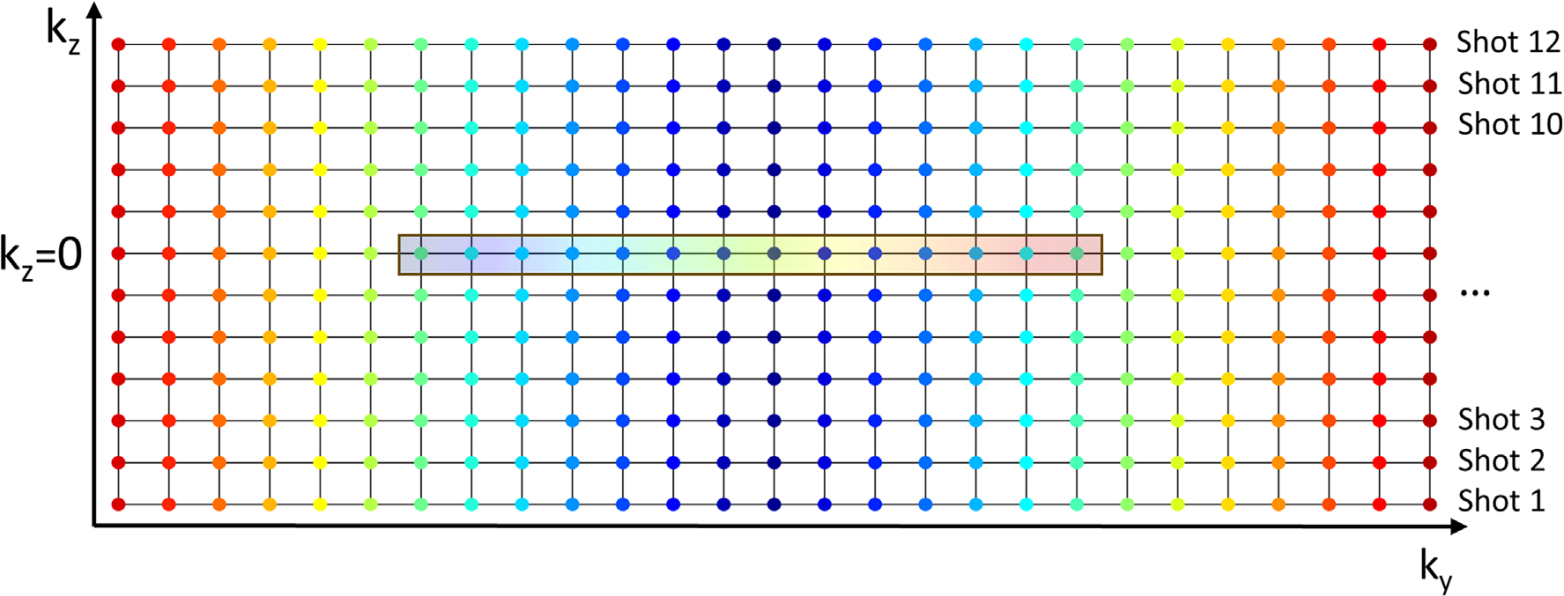
K‐space sampling for the 3D diffusion acquisition and 2D phase navigator. Each colored circle represents the acquisition of a frequency‐encoded line (kx) in the 3D diffusion acquisition, with centric ordering in the phase‐encoding (ky) direction (for each shot, blue‐colored k‐space points are acquired earlier than red‐colored k‐space points). Each shot of the 3D diffusion acquisition acquires all ky samples for a single partition‐encoding step (i.e., one row). The rectangle at the centre of k‐space indicates the k‐space samples acquired with the 2D navigator, which are acquired with linear encoding and are identical for every shot.

The sequence is similar in its design and principles to the DW turbo‐STEAM^56^ and diffusion‐prepared stimulated echo turboFLASH^53^ sequences and enables a 3D multi‐shot diffusion‐prepared acquisition with reduced FOV and explicit 2D phase navigation. The sequence (Figure 1) consists of a twice‐refocused DP scheme, paired with a centric‐encoded 3D GRE readout, followed by a linearly encoded 2D phase correction navigator. Each imaging shot acquires a single partition of the 3D acquisition, that is, in each imaging shot all the in‐plane phase encoding steps are acquired for one partition‐encoding step. The 2D phase navigator is acquired with the same excitation pulse but without any partition encoding and with lower resolution in the phase‐encoding direction. The k‐space acquisition for 3D diffusion imaging and 2D phase navigation is illustrated in Figure 2. The DP is preceded by a chemical shift‐selective fat‐saturation pulse. The tip‐down pulse in the DP is applied simultaneously with a slab‐selective gradient in the phase‐encoding direction, thereby restricting the tip‐down excitation in the DP module to water protons within a reduced FOV. The tip‐down excitation is followed by a pair of bipolar diffusion gradients, each surrounding a 180° adiabatic hyperbolic secant refocusing pulse. A magnitude stabilizer approach is used to avoid phase‐offset–induced magnitude errors and convert these into conventional intershot phase errors. Finally, a nonselective 90° tip‐up pulse is applied. It is worth noting that whereas this acts as a tip‐up pulse for the inner‐volume magnetization, it acts as a tip‐down pulse for the unprepared outer‐volume magnetization and any recovered fat magnetization. A spoiler gradient is executed immediately after the DP to dephase any remaining transverse magnetization, including outer volume and/or fat protons. The original phase of the spins is restored by a magnitude stabilizer rewinder gradient, which is played out after each RF readout pulse and importantly also acts as a spoiler gradient for the outer volume and fat magnetization.

The remaining inner‐volume intershot phase variations are corrected with the 2D phase navigator in the reconstruction: a Hamming filter is applied to each phase navigator in k‐space for each receiver coil to reduce Gibbs ringing. A 2D inverse Fourier transform is then applied to both the Hamming‐filtered phase navigator data and to each diffusion‐prepared imaging shot for each receiver coil to obtain 2D (complex) images, *N*_*i*_(*x*,*y*) and *S*_*i*_(*x*,*y*), for the *i*‐th navigator and diffusion‐prepared shot, respectively. The Hamming‐filtered phase navigator data are then normalized to have a magnitude equal to 1. A complex conjugate multiplication of the two respective datasets is then performed in image space.^13^ The navigator normalization and phase correction of *S*_*i*_(*x*,*y*) are summarized in the following equation:

$$S_{i,\text{corr}}\left(x,y\right)=S_{i}\left(x,y\right)\;\frac{N_{i}^{*}\left(x,y\right)}{\left|N_{i}\left(x,y\right)\right|},$$

where *S*_*i*_, _*corr*_(*x*, *y*) is the result of the phase correction of *S*_*i*_(*x*, *y*), and N*_i_(*x*, *y*) and |*N*_*i*_(*x*, *y*)| are the complex conjugate and the magnitude of *N*_*i*_(*x*, *y*), respectively.

Finally, a Fourier transform is applied to produce the phase‐corrected image.^18^ The reconstruction was implemented inline in the vendor reconstruction software (Syngo MR E11P, Siemens Healthineers AG, Erlangen, Germany).

### 2.2 Imaging studies

All imaging was performed at 3 T (Biograph mMR, Siemens Healthineers AG). Phantom imaging was acquired with a 16‐channel head and neck coil, and imaging in vivo was acquired with a 32‐channel spine array and posterior section of a 16‐channel head and neck coil.

#### 2.2.1 Phantom study

A National Institute of Standards and Technology (NIST)–traceable diffusion phantom (Caliber MRI, Boulder, CO) with six vials containing varying concentrations of polyvinylpyrrolidone (PVP) and water was used in the phantom experiments.

The phantom was imaged with the proposed sequence and a standard 2D DW‐SS‐EPI sequence to evaluate the accuracy of ADC estimates. The diffusion sequences were acquired with matched parameters where possible, as detailed in Table 1. Additional parameters for the proposed sequence include number of navigator lines: 14, one dummy scan for magnetization preparation, and 4π intravoxel phase dispersion for the magnitude stabilizer, and the tip‐down pulse in the DP is a 4 ms Hamming‐filtered sinc pulse with time‐bandwidth product = 12.

**TABLE 1:** Acquisition parameters for phantom and in vivo studies.

	Phantom		In Vivo		
	Proposed	SS‐EPI	Proposed	SS‐EPI	RESOLVE
b‐values (s/mm^2^)	0, 500		0, 500	0, 500	0, 500
Averages	1, 1	1, 5	1, 5		
In‐plane resolution (mm × mm)	2.1 × 2.1		2.1 × 2.1		
Slice thickness (mm)	4		4		
Number of slices	40		8		
Partial Fourier	None		None		
FOV read × FOV phase (mm^2^)	200 × 75	430 × 263	200 × 50	430 × 263	220 × 112
TR (ms)	2000^a^	15600^b^	2000^a^	4200^b^	2800^b^
Echo spacing (ms)	4.21	0.66	4.21	0.66	0.38
TE (ms)	2.36	79	2.36	79	62
Diffusion prep time (ms)	65	N/A	65	N/A	N/A
Excitation flip angle (°)	10	90	10	90	90
Phase‐encode lines per shot	41	63	27	63	48
Readout segments					5
Acceleration	None	GRAPPA 2	None	GRAPPA 2	GRAPPA 2
Fat suppression	FatSat^95^	STIR^96^	FatSat^95^	STIR^96^	FatSat with gradient reversal^97^
Receiver bandwidth (Hz/Px)	410	2025	410	2025	945
Phase‐encode bandwidth (Hz/Px)		24		24	53
Phase oversampling	10%	0%	10%	0%	75%
Slice oversampling	0%		50%		
Acquisition time (min:s)	5:22	5:12	6:56	1:26	3:54

Abbreviations: FatSat, fat saturation; STIR, short tau inversion recovery; RESOLVE, readout‐segmented EPI diffusion sequence; SS‐EPI, single shot EPI.

^a^ For the proposed sequence, TR is the time between successive shots (partition encodings) of the 3D acquisition.

^b^ For EPI and RESOLVE sequences, TR is the time between successive excitations of the same slice.

ADC maps were calculated for both sequences using mono‐exponential fitting of the signal from the b0 and b500 trace‐weighted images.

#### 2.2.2 In vivo evaluation

Five subjects (four male, one female, mean age 30 ± 4 years) were prospectively recruited for the study. This study was approved by the institutional ethics committee (REMAS 8700), and written informed consent was obtained from all participants for the scan and inclusion in this study. The cervical spinal cord was imaged with the proposed sequence, a standard 2D DW‐SS‐EPI sequence, clinical 2D multi‐shot readout‐segmented EPI diffusion sequence (RESOLVE),^13^ and 2D T_2_‐weighted TSE sequence as anatomical reference. The sequence parameters for all diffusion sequences are detailed in Table 1. Additional parameters for the proposed sequence were matched to the phantom study.

ADC maps were calculated for all sequences using mono‐exponential fitting of the signal from the b0 and b500 trace‐weighted images. For all diffusion sequences, a ROI in the upper cervical spinal cord was manually delineated on the ADC map on the imaging slice that captured the centre of the spinal cord. For each volunteer and each diffusion sequence, the ADC values were estimated as the mean ADC in the ROI. For statistical analysis, a Wilcoxon signed‐rank test was performed between the ADC values of the proposed sequence and the ADC values of DW‐SS‐EPI and RESOLVE, with a *p*‐value <0.05 indicating a significant difference.

### 2.3 Qualitative assessment

The images were assessed qualitatively independently by two radiologists with >20 and >5 years of experience in MRI. Overall image quality (1 = nondiagnostic, 2 = poor, 3 = fair, 4 = good, 5 = excellent), perceived distortion (1 = major distortion resulting in nondiagnostic image quality, 2 = major distortion but not limiting diagnosis, 3 = minor distortion but not limiting diagnosis, 4 = no distortion), and perceived SNR (1 = poor, 2 = fair, 3 = good, 4 = very good, 5 = excellent) were assessed for each subject/acquisition.

## 3 RESULTS

### 3.1 Phantom study

**FIGURE 3:**
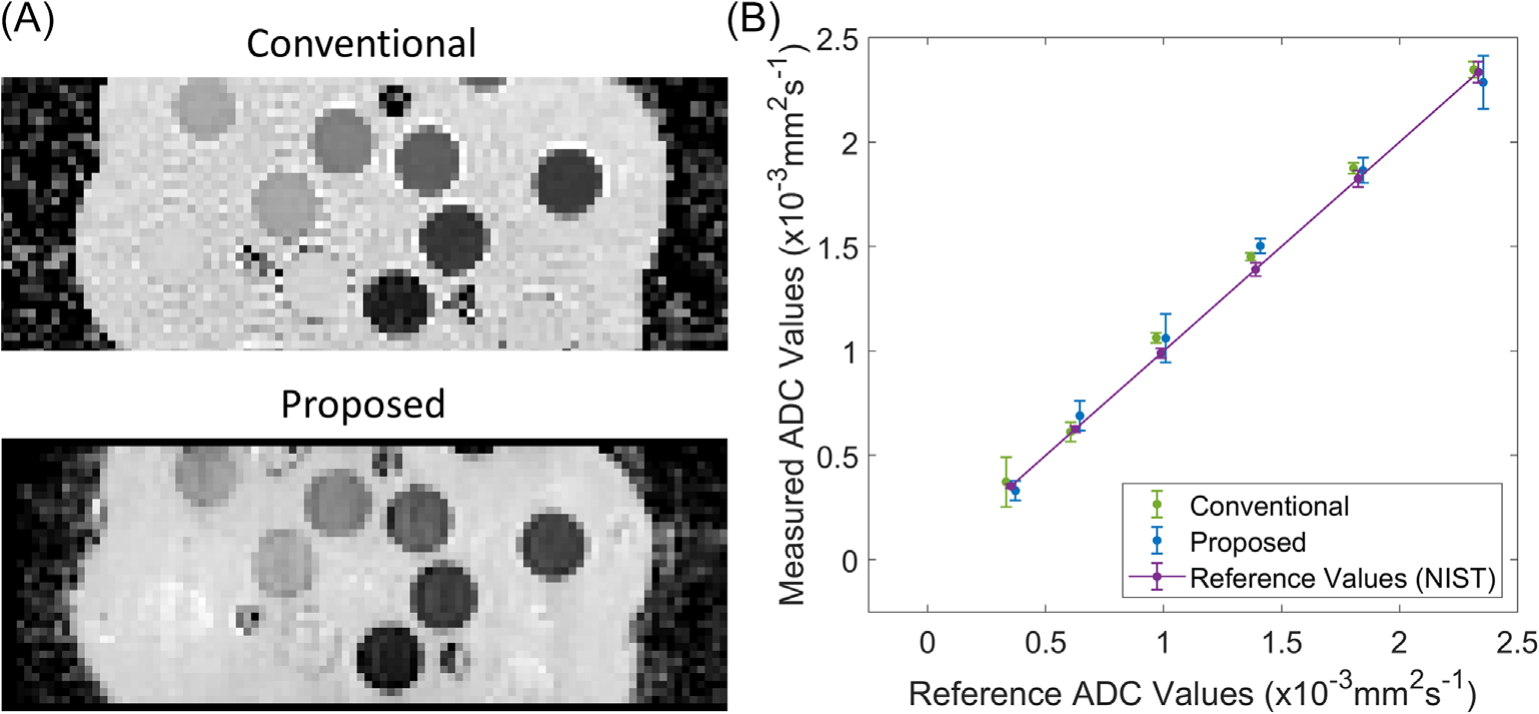
Diffusion phantom measurements. (A) ADC maps calculated using the conventional SS‐EPI diffusion sequence (top) and the proposed sequence (bottom). (B) Plot comparing the mean and SDs of ADC values in six vials measured with the conventional SS‐EPI sequence (green) and the proposed sequence (blue) against the reference values (purple) provided by the manufacturer. Proposed and conventional measurements are slightly offset in the x‐direction for clarity. SS‐EPI, single shot EPI.

ADC maps in the diffusion phantom calculated for the proposed sequence and the conventional DW‐SS‐EPI sequence are shown in Figure 3A, and a plot of the obtained ADC values using both sequences against the reference ADC values provided by the manufacturer of the phantom is shown in Figure 3B. There was a good correlation between the ADC values obtained from the proposed sequence and the reference ADC values (slope and intercept of linear fit: 0.98 and 0.06 × 10^−3^ mm^2^/s R^2^ = 1.0, RMS error = 0.05 × 10^−3^ mm^2^/s). A similar correlation was observed between the ADC values obtained from the DW‐SS‐EPI sequence and the reference ADC values (slope and intercept of linear fit: 1.01 and 0.03 × 10^−3^ mm^2^/s, R^2^ = 1.0, RMS error = 0.04 × 10^−3^ mm^2^/s).

### 3.2 In vivo evaluation

**FIGURE 4:**
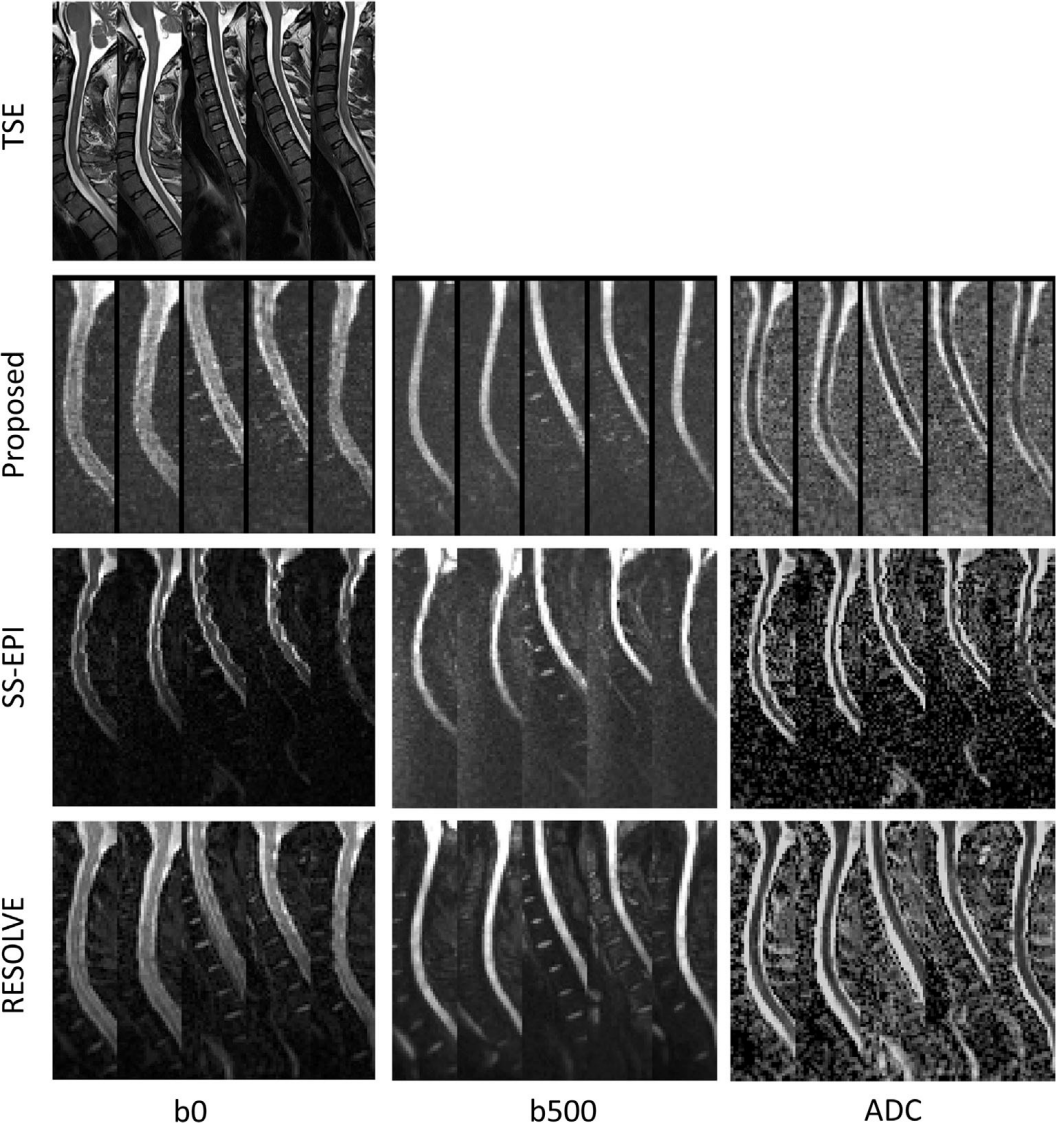
Example images acquired in vivo: T_2_‐weighted TSE (top row), b0 (left column); b500 trace‐weighted images (middle column); and ADC maps (right column) for the proposed sequence, DW‐SS‐EPI, and RESOLVE for all five volunteers. It is noted that the contrast between the bone marrow and intervertebral discs is lower for the proposed sequence compared to the reference sequences, which may be attributed to complex averaging of b500 images with the proposed sequence, leading to elevated ADC values in low SNR regions (such as bone marrow). DW, diffusion‐weighted; SS‐EPI, single shot EPI; RESOLVE, readout‐segmented EPI diffusion sequence; TSE, turbo spin echo.

**FIGURE 5:**
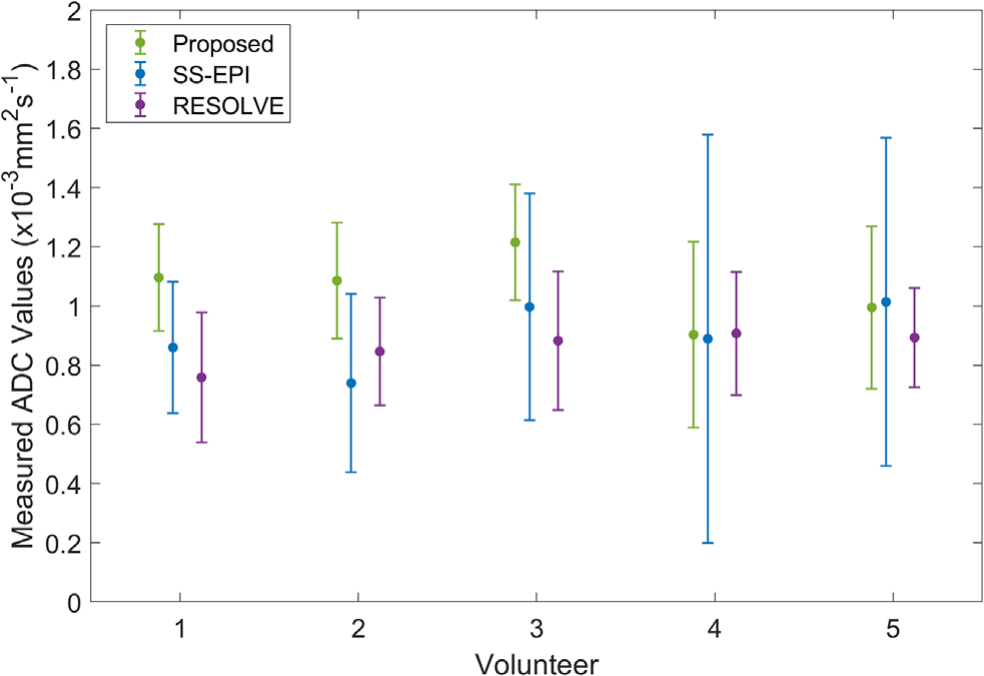
Mean and SDs of measured ADC values in ROIs in the upper cervical spine for the proposed sequence, DW‐SS‐EPI, and RESOLVE in the five healthy volunteers. DW, diffusion‐weighted; SS‐EPI, single shot EPI; RESOLVE, readout‐segmented EPI diffusion sequence; ROI, region of interest.

The left column of Figure 4 shows one slice from the T_2_‐weighted TSE and b0 images for the proposed sequence, DW‐SS‐EPI, and RESOLVE for all five volunteers. The middle and right columns of Figure 4 show the same slice from the b500 trace‐weighted images and calculated ADC maps, respectively. Contour plots of the CSF and cord, delineated on the TSE images, are shown overlaid on the b0 and b500 images in Supporting Information Figure S1 to better highlight the distortion properties of each sequence. Figure *5* shows the estimated ADC values in the ROI for each volunteer and diffusion sequence. There were no significant differences in ADC estimates measured with the proposed sequence (1.06 ± 0.12 × 10^−3^ mm^2^/s) compared with DW‐SS‐EPI (0.90 ± 0.11 × 10^−3^ mm^2^/s, *p* = 0.19) or RESOLVE (0.86 ± 0.06 × 10^−3^ mm^2^/s, *p* = 0.13). The mean image quality, perceived distortion, and perceived SNR scores from the qualitative assessment are shown in Table 2.

**TABLE 2:** Mean scores from qualitative assessment by two expert readers of b500 images acquired in vivo in five healthy volunteers.

	DW‐SS‐EPI	RESOLVE	Proposed
Image quality	1.5 ± 0.4	3.8 ± 0.3	3.3 ± 0.3
Perceived distortion	1.5 ± 0.4	3.3 ± 0.4	3.6 ± 0.2
Perceived SNR	1.8 ± 0.4	3.7 ± 0.3	3.3 ± 0.3

## 4 DISCUSSION

In this study, a 3D RFOV diffusion‐prepared GRE sequence is presented for distortion‐free diffusion imaging. The sequence was tested in vivo for spinal cord DWI and compared to standard DW‐SS‐EPI and to a clinical multi‐shot DWI sequence with reduced distortions (readout‐segmented EPI, RESOLVE^13^), which has been successfully applied for spine DWI (among other applications) in previous studies.^70^, ^71^ The proposed sequence demonstrated improved image quality, improved perceived SNR, and reduced perceived distortion compared to DW‐SS‐EPI, whereas all measures were comparable against RESOLVE. Furthermore, all images acquired in vivo demonstrated effective suppression of outer‐volume and fat signal. The obtained ADC values in a phantom and in the upper cervical spine in healthy volunteers were consistent with those obtained using the reference sequences (DW‐SS‐EPI for the phantom scans; DW‐SS‐EPI and RESOLVE for the study in vivo).

The RFOV approach is a key element of the sequence design because this reduces the readout duration and enables acquisition of all the lines of a partition within a single shot while maintaining sufficient signal for the navigator acquisition, which is acquired after the imaging readout and permits a straightforward phase correction.

The efficacy of the novel RFOV method introduced in this study can be attributed to the outer‐volume signal suppression achieved using a slice‐selective/nonselective tip‐up/tip‐down DP, combined with a magnitude stabilizer approach, and this combination is the main contribution of this paper. The outer volume is not excited by the tip‐down pulse of the DP. Therefore, the “tip‐up” pulse and subsequent spoiler gradient serve to saturate the outer volume magnetization before the readout. On its own, this outer‐volume suppression may be insufficient because small inefficiencies in the outer‐volume saturation or regrowth of outer‐volume magnetization over the course of the readout may still significantly contaminate the low‐signal diffusion‐prepared magnetization.^54^ Therefore, it is important to also recognize the contribution of the magnitude stabilizer rewinder gradient to the suppression of outer volume signal: The dephasing gradient of the magnitude stabilizer in the DP, applied before the tip‐up pulse, does not influence magnetization in the outer volume because it has not been excited by the tip‐down pulse, and thus the magnitude stabilizer rewinder gradient serves to spoil any signal from outer‐volume magnetization that recovers during the course of the readout.

ADC mapping for the proposed sequence was performed using mono‐exponential fitting, as proposed in the stimulated echo turboFLASH sequence.^53^ This is possible because the magnitude stabilizers spoil the transverse magnetization, and therefore the net longitudinal magnetization is zero after the tip‐up pulse. Therefore, after one dummy shot, the steady‐state longitudinal magnetization available before the tip‐down pulse is independent of the diffusion weighting, that is, it will be the same independently of the used b‐value (but still dependent on T_1_ recovery). In addition, the regrowing longitudinal magnetization does not contribute to the stimulated echo pathway. These factors make the ADC estimation more straightforward for this sequence than methods using analytical signal fitting or dictionary matching with a forward simulation of the sequence in which prior knowledge of T_1_ and T_2_ relaxation times is required.^48^, ^51^

Fat suppression is critical in EPI‐based diffusion due to chemical shift–related displacement in the low‐bandwidth phase‐encoding direction.^72^ In the proposed sequence, effective fat suppression is also desirable to achieve optimal image quality. Chemical shift–related displacement of fat in the phase‐encoding direction when applying the slab‐selective tip‐down pulse may lead to fat signal outside of the reduced FOV being excited by the tip‐down pulse and folding back in the reduced FOV. This effect may be exacerbated due to the tip‐down pulse being a relatively long 90° pulse exciting a larger slab thickness than typical 2D slices, leading to a low slab‐selection gradient strength. Unsuppressed fat signal may also be detrimental for the 2D phase navigator because the 2D navigator is essentially a projection of a 3D slab and the projection of surrounding fat tissue might overlap with the ROI, resulting in a less reliable phase estimation. Such contamination in the diffusion‐weighted images and in the phase navigator is particularly problematic due to the low ADC of fat, resulting in hyperintense signal compared to the tissue of interest. Furthermore, radiologists are more familiar with reading fat‐suppressed DWI because this is the standard in the clinic. Fat signal was successfully suppressed in our spinal cord experiments in a similar manner, as described above for outer‐volume signal because the fat saturation pulse, when effective, ensures that fat signal is also not excited by the tip‐down pulse of the preparation module. Whereas the fat signal was successfully suppressed in all images acquired in vivo in this study, there may be cases/anatomies where the chemical shift‐selective saturation fails, especially in regions with high B_0_ inhomogeneity and magnetic susceptibility gradients. However, this sequence could be combined with a multi‐echo Dixon readout for fat–water separation to achieve a more robust fat suppression.^33^, ^73^, ^74^

From a sequence design perspective, the proposed approach builds on principles proposed in DW turbo‐STEAM^56^, ^58^ and diffusion‐prepared stimulated echo turboFLASH^53^ to enable a 3D multi‐shot reduced FOV acquisition. A reduced FOV version of the DW turbo‐STEAM sequence was previously proposed and applied for diffusion imaging of the spine^75^; however, this sequence was restricted to a single‐shot 2D acquisition and used slice‐selective 90° pulses in orthogonal directions to achieve FOV reduction, whereas the proposed sequence uses a combination of slice‐selective and nonselective tip‐down/‐up pulses. A 2D multi‐shot self‐navigated extension to DW turbo‐STEAM was proposed using a radial trajectory and a nonlinear reconstruction algorithm^58^; however, the proposed sequence combines a 3D acquisition with a Cartesian trajectory, 2D phase navigators, and reduced FOV DP.

Compared to diffusion‐prepared TSE sequences,^60^, ^61^, ^62^, ^63^ diffusion‐prepared GRE sequences offer an alternative for non‐EPI diffusion imaging with a much lower SAR deposition burden. This is because SAR is proportional to the square of the RF flip angles,^76^ and the proposed sequence uses low flip angle RF excitation pulses (if we consider the oversimplified situation where pulse shapes and durations are the same, and for 180° TSE refocusing pulses, the energy of the 10° excitation pulse in the proposed sequence amounts to 0.3% of the energy of the TSE refocusing pulse). This could be beneficial especially given that the DP requires the use of a pair of SAR‐intensive adiabatic refocusing pulses at 3 T to achieve robustness to B_1_ (and B_0_) inhomogeneity effects.^77^

A further difference between the proposed sequence and diffusion‐prepared TSE is that whereas TSE readouts are affected by T_2_ decay along the echo train, the proposed sequence with a centric trajectory is affected by the T_1_ decay of the stimulated echoes.^75^, ^78^ Whereas both may lead to blurring artifacts, T_1_ relaxation times are typically longer than T_2_ relaxation times, and the achieved echo spacings for GRE readouts are usually shorter than for TSE readouts. Thus, it is expected that the blurring effects related to T_1_ decay are less pronounced than those due to T_2_ decay. However, T_1_‐related blurring may be observed for species with short T_1_ relaxation times such as unsuppressed fat or tissue after contrast agent injection.

Despite the advantages of increased Fourier averages due to partition encoding with a 3D acquisition and the lower receiver bandwidths per pixel, the SNR efficiency of the proposed sequence is relatively low compared to the DW‐SS‐EPI and RESOLVE sequences. This is in part due to the inherent 50% signal loss incurred by all diffusion sequences, which employ magnitude stabilizers due to the creation of a stimulated echo.^79^ In addition, the use of a low flip angle GRE readout further reduces the expected signal compared to a spin echo EPI sequence. Two‐dimensional diffusion‐weighted sequences also benefit from slice interleaving, which increases the scan efficiency compared to the proposed 3D sequence, where time is needed for magnetization recovery after each GRE shot, as evidenced by the shorter scan times of the 2D DW‐SS‐EPI and RESOLVE sequences. However, other factors impacting the SNR efficiency of the proposed sequence are application‐specific. For example, application of this sequence to regions requiring an increased number of partitions could offer an increase in SNR efficiency because signal averaging of the high b‐value acquisitions could be traded for Fourier averaging by increasing the number of partition‐encoding steps, resulting in preserved SNR and scan time with increased coverage. Another application‐specific factor impacting SNR is the shot‐to‐shot TR. A relatively long shot‐to‐shot TR (2 s) was used for the proposed sequence in this study to ensure sufficient longitudinal magnetization recovery and a high steady‐state signal in the spinal cord. However shorter shot‐to‐shot TRs could be achieved and could be beneficial for the study of regions with short T_1_ relaxation times, resulting in improved SNR efficiency while achieving much lower SAR deposition than for diffusion‐prepared TSE.

Our spinal cord experiments were performed using thin slabs (32 mm) where 2D phase correction is adequate.^80^ For thicker slabs, the 2D navigator phase correction could be less accurate due to through‐slab phase variations,^81^ and in these cases it may be necessary to implement an accelerated 3D navigator for robust phase correction.^65^, ^82^, ^83^, ^84^, ^85^ Furthermore, alternative self‐navigated phase correction approaches for 3D multi‐shot diffusion imaging have been proposed^25^ and could be used to replace the 2D phase navigators.

A limitation of the proposed technique is that it may be sensitive to imperfections in the tip‐down and tip‐up pulses of the DP. B_1_ inhomogeneities, which are more pronounced at 3 T compared to 1.5T,^86^ affect both pulses and may lead to a decrease in the available diffusion‐prepared magnetization within the reduced FOV. We would like, however, to note that this limitation is not specific to this sequence and applies to diffusion‐prepared sequences using standard nonselective tip‐down and tip‐up pulses. A further effect affecting the nonselective tip‐up pulse is that B_1_ inhomogeneities outside of the reduced FOV would also affect the amount of residual unprepared longitudinal magnetization from the outer volume at the end of the DP, which may increase sensitivity to imperfections in the spoiling using magnetization stabilizer rewinders. The performance of the FOV reduction may also depend on the slice profile of the tip‐down pulse, and we have mitigated this in the proposed sequence by applying a small amount of phase oversampling and using a tip‐down pulse with a high time‐bandwidth product.

A further limitation of the technique is that the required phase FOV may be subject‐dependent, especially for imaging the spinal cord, for which adaptation of the phase FOV may be needed depending on spine curvature. This would lead to variability in the achieved shot durations for the proposed sequence and hence variability in the benefits of the technique such as reducing T_1_ decay across the shot. In addition, the proposed sequence is inherently limited in its use to organs in which a reduced FOV approach is suitable and therefore has less applicability than other multi‐shot DWI techniques with reduced distortion such as readout‐segmented EPI.^13^

Pulsatile cord and CSF motion are known to introduce artifacts when imaging the spinal cord,^87^ leading to signal voids in diffusion‐weighted images due to incoherent intravoxel motion,^88^ even for single‐shot diffusion EPI sequences.^89^ These signal voids pose a challenge for accurate and reproducible ADC mapping along the spinal cord,^87^, ^89^ which may give rise to some of the variability in ADC values observed in vivo in the current study (Figure 5). Similarly to a previous study,^90^ we have therefore focused on the upper cervical spine for our ADC mapping study because it is less susceptible to cardiac and respiratory motion. Whereas cardiac or pulse triggering have also been used to mitigate against the pulsatility of the spinal cord,^91^ this is not desirable in the sequence presented because it would introduce a variable magnetization recovery delay. An alternative solution is to use optimized diffusion gradient waveforms, such as motion‐compensated diffusion gradients to minimize the effect of bulk motion^50^, ^89^; however, these may lead to prolonged DP times and more pronounced signal loss due to T_2_ relaxation.

This is a proof‐of‐concept study, and thus we limited our exploration of this technique to the cervical spinal cord in a small number of healthy volunteers. Other applications with restricted imaging regions could also benefit from this RFOV technique, such as the prostate, cervix, lumbosacral/sciatic, and optic nerves. Further testing of this sequence is required in regions with increased off‐resonance and for radiotherapy applications in which geometric fidelity requirements are stringent. In our sequence evaluation, we did not include a comparison with reduced FOV SS‐EPI diffusion sequences, which are expected to exhibit reduced distortions compared to standard SS‐EPI sequences, as shown in previous spinal cord imaging studies.^38^, ^40^, ^90^ The phase FOV of the DW‐SS‐EPI acquisition was not optimized for acquisition in the cervical spine; however, DW‐SS‐EPI is not the gold standard clinical sequence for spinal cord DWI, and pronounced distortions would be expected to persist.^90^ The sequence was acquired with a relatively large slice thickness (4 mm) to achieve acceptable SNR; therefore, more advanced reconstruction techniques such as deep learning–based reconstruction for diffusion imaging^92^, ^93^, ^94^ could be considered to enable an isotropic resolution 3D scan with diagnostic image quality without a significant scan time penalty.

## 5 CONCLUSION

In this work, we proposed a novel 3D reduced FOV, diffusion‐prepared GRE sequence and showed initial results for feasibility of spinal cord diffusion imaging in healthy volunteers. A clinical study in patients is warranted to further evaluate the potential of this technique.

## CONFLICT OF INTEREST STATEMENT

Dr Sarah McElroy and Dr Raphael Tomi‐Tricot are employed by Siemens Healthineers. The rest of the authors do not have any conflicts of interest related to this work.

## Supporting information


**Figure S1.** Example in‐vivo images of the T_2_‐weighted TSE (top row), b0 (left, bottom 3 rows) and b500 trace‐weighted images (right, bottom 3 rows) for the proposed sequence, DW‐SS‐EPI and RESOLVE for all 5 volunteers with contour plots of CSF and spinal cord overlaid on the images to better highlight the distortion properties of each sequence.
