# Supplementary material for: 3D distortion‐free, reduced FOV diffusion‐prepared gradient echo at 3 T
Source: Magn Reson Med. 2024 Oct 27;93(4):1471–83. doi: 10.1002/mrm.30357 (PMC11782725; doi:10.1002/mrm.30357)
Supplement: Supplementary file 1 — Figure S1. Example in‐vivo images of the T2‐weighted TSE (top row), b0 (left, bottom 3 rows) and b500 trace‐weighted images (right, bottom 3 rows) for the proposed sequence, DW‐SS‐EPI and RESOLVE for all 5 volunteers with contour plots of CSF and spinal cord overlaid on the images to better highlight the distortion properties of each sequence. [file MRM-93-1471-s001.docx]

Supporting Information


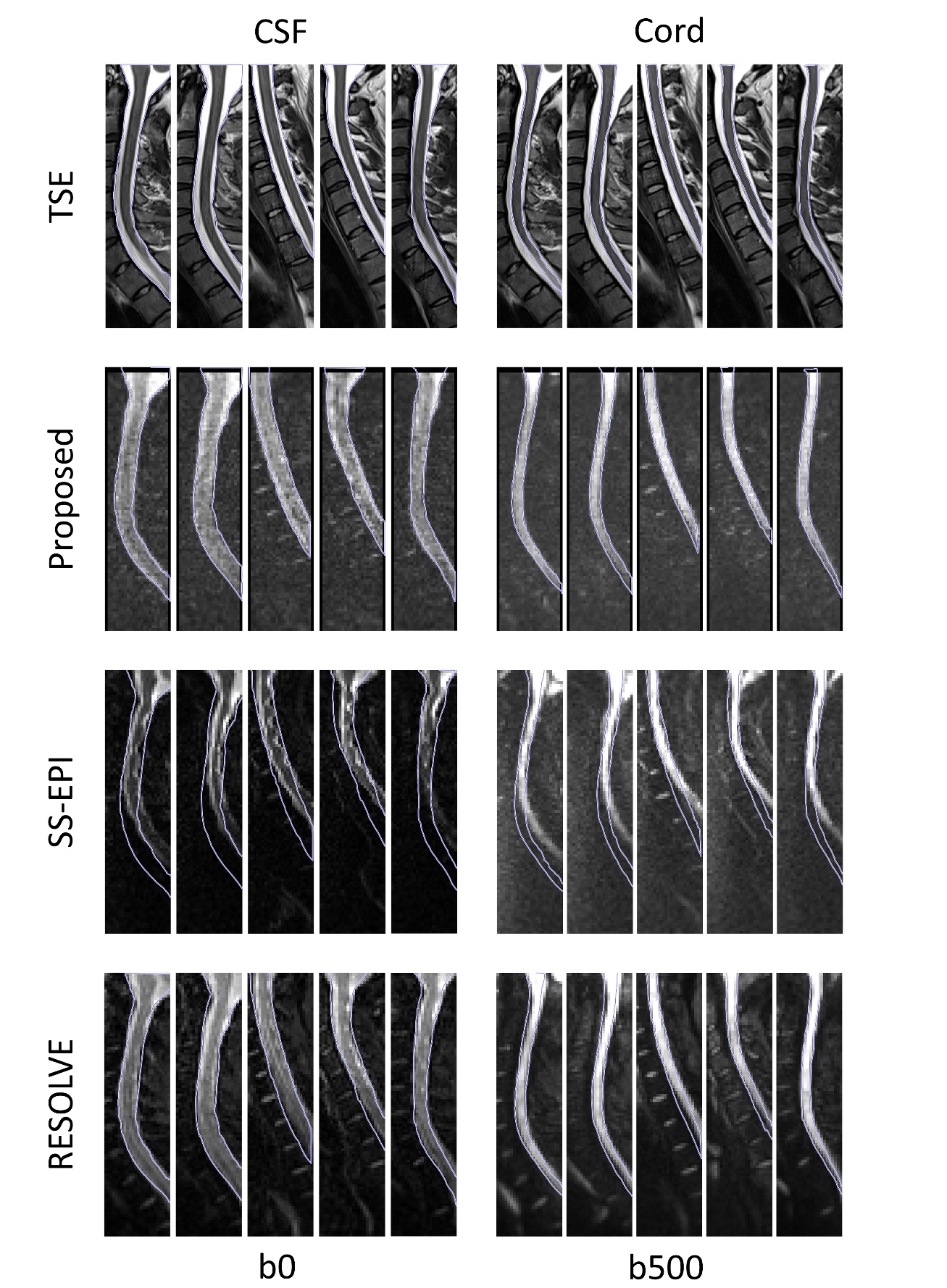


Figure S1: Example in-vivo images of the T2-weighted TSE (top row), b0 (left, bottom 3 rows) and b500 trace-weighted images (right, bottom 3 rows) for the proposed sequence, DW-SS-EPI and RESOLVE for all 5 volunteers with contour plots of CSF and spinal cord overlaid on the images to better highlight the distortion properties of each sequence
